# Top-down and bottom-up modulation in processing bimodal face/voice stimuli

**DOI:** 10.1186/1471-2202-11-36

**Published:** 2010-03-11

**Authors:** Marianne Latinus, Rufin VanRullen, Margot J Taylor

**Affiliations:** 1 Université de Toulouse, UPS, CNRS, Centre de recherche Cerveau et Cognition, Toulouse, France; 2 Centre for Cognitive Neuroimaging (CCNi) and Department of Psychology, University of Glasgow, Glasgow, G128BQ, UK; 3 Diagnostic Imaging and Research Institute, Hospital for Sick Children, University of Toronto, Toronto, Ontario, M5G1X8, Canada

## Abstract

**Background:**

Processing of multimodal information is a critical capacity of the human brain, with classic studies showing bimodal stimulation either facilitating or interfering in perceptual processing. Comparing activity to congruent and incongruent bimodal stimuli can reveal sensory dominance in particular cognitive tasks.

**Results:**

We investigated audiovisual interactions driven by stimulus properties (bottom-up influences) or by task (top-down influences) on congruent and incongruent simultaneously presented faces and voices while ERPs were recorded. Subjects performed gender categorisation, directing attention either to faces or to voices and also judged whether the face/voice stimuli were congruent in terms of gender. Behaviourally, the unattended modality affected processing in the attended modality: the disruption was greater for attended voices. ERPs revealed top-down modulations of early brain processing (30-100 ms) over unisensory cortices. No effects were found on N170 or VPP, but from 180-230 ms larger right frontal activity was seen for incongruent than congruent stimuli.

**Conclusions:**

Our data demonstrates that in a gender categorisation task the processing of faces dominate over the processing of voices. Brain activity showed different modulation by top-down and bottom-up information. Top-down influences modulated early brain activity whereas bottom-up interactions occurred relatively late.

## Background

The ability to integrate information from several sensory modalities is a vital skill of the human brain, as information we receive from the external world is often multimodal. Although there has been a recent surge of research focusing on the processing of multimodal information, our knowledge of the neural substrates underlying this ability for complex stimuli in humans is still limited.

Researchers have used two main paradigms to investigate multimodal processing. One is designed to assess the perceptual gain of multisensory inputs by comparing the behaviour and the neural activity evoked by multimodal and unimodal inputs [1,2]. The other paradigm assesses the competition between senses using bimodal stimuli which could be either congruent or incongruent; using incongruent stimuli can reveal the existence of a cross-modal bias [3]. These two approaches yield different information: the first determines the advantages and limits of multimodality, while the second provides information on sensory dominance and its influence on task performance. The present study investigates sensory competition or dominance in the processing of gender in bimodal face/voice stimuli.

Sensory dominance has been largely studied in terms of spatial localisation or temporal discrimination. The research approach of comparing congruent and incongruent bimodal stimuli has demonstrated that the influence of the senses is asymmetric and task-dependent. For example, in ventriloquism, the visual-spatial information biases the localisation of the source of auditory information toward the source of visual information [4-6]. The localisation of a visual stimulus is however, almost unaffected by simultaneous discordant auditory information [4]. In contrast, in the temporal domain, the auditory modality dominates the visual, i.e. when subjects judge temporal aspects of a stimulus (frequency of occurrence, temporal frequency, etc.), auditory stimuli modulate perceived information in the visual modality [7-9]. These results suggest that in the spatial domain, vision dominates audition, while in the temporal domain, the reverse is true [10]. Using emotional faces and voices, it has been demonstrated that a static face alters the perception of vocal emotion even when the task required ignoring the face [3,11]. One aim of the present study was to determine if sensory dominance could be observed in the processing of faces and voices, i.e. is the influence of one sensory modality on the other equivalent or symmetrical in the perception of gender? To this purpose, we manipulated attention through task demands on congruent and incongruent face/voice stimuli.

Neural correlates of multimodal processing have been investigated using fMRI, PET and ERPs, with results showing that bimodal processing was task-sensitive [12]. As in the behavioural literature, various approaches have been used to study neural mechanisms underlying multimodal processing. Comparing the brain activity for bimodal stimuli to the sum of activity for unimodal stimuli (e.g., AV - (A+V)) revealed that congruent bimodal stimuli enhanced brain activity either in sensory-specific cortices [1,13,14] or in brain regions described as heteromodal [15]. The timing of this bimodal activation was very rapid, affecting brain processing within 40 ms [1,16-18]. Even with more biological stimuli (sounds and pictures of animals), early interactions between visual and auditory processing were seen on the visual N1 component (~150 ms) [19].

Investigations of higher-level multimodal processing critical to human social interactions (faces and voices) have been less common, with most studies on face and voice integration focussed on speech processing. The interaction between visual and auditory stimuli in the speech domain is classically demonstrated by the McGurk effect [20]. As seen with simple bimodal object and spatial processing, there is a behavioural advantage of bimodal redundant speech [21]. Audiovisual integration of faces and voices has also been shown in non-human primates, as monkeys are able to match a face and a certain vocalisation [22], demonstrating its wider application to other social species. The small literature on face/voice interactions in a non-verbal context is largely focussed on emotional processing [23-25]. Emotion expression protocols have also been used with monkeys, as Parr (2004) showed, in a match-to-sample task, a modality preference depending on the expression to be matched [26]. Bidirectional interference in processing has been demonstrated with incongruent emotional voices and faces [27] suggesting no sensory dominance in the processing of emotions. Congruent emotional faces and voices enhance the auditory N1 [11,25]; yet, in a bimodal speech perception study, the opposite was demonstrated, a reduced N1 to congruent bimodal stimuli [21].

Although face/voice associations to extract non-speech information have been rarely studied, there is a wealth of face and voice processing studies in unimodal paradigms. A large literature provides evidence that faces are processed through a distributed and hierarchical network [28]; neurophysiological studies provide latencies for the different stages of face processing. The N170 component is sensitive to a range of manipulations of faces [29-32] suggesting that it reflects automatic face processing [33,34]. Earlier components have also been reported to be face-sensitive [35,36].

Comparable studies have been completed with voices, often referred to as 'auditory faces' due to the similarity of information carried by faces and voices [37,38], and have revealed that the processing of non-speech information of voices involved structures located along the right superior temporal sulcus. There are few ERP studies comparing voices to other auditory stimuli. Two papers report a positive deflection 320 ms after stimulus onset that is larger to voices than to musical instrument stimuli, labelled the Voice Selective Response (VSR) [39,40]. A recent study comparing voices to various non-vocal sounds suggests that the voice/non-voice discrimination could occur earlier, in the latency range of the auditory P2, 160-240 ms [41]. The processing of faces and voices seems thus to draw on specialised and distinct brain regions and to have distinct temporal profiles.

**Figure 1:**
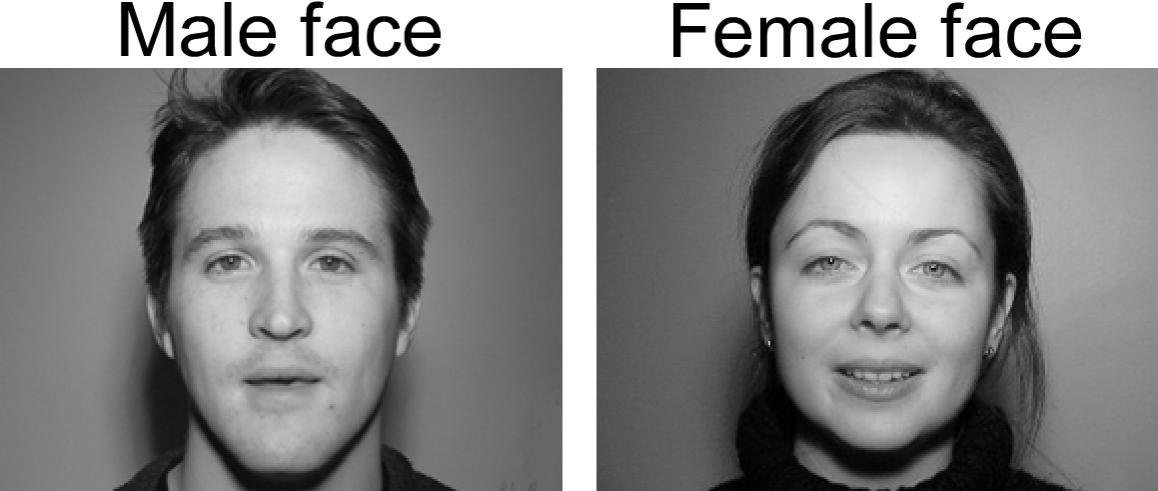
**Examples of face stimuli**.

The integration of information from faces and voices is a crucial skill that is essential for normal social interactions. Determining how cross-modal processing of faces and voices occurs will contribute significantly to our understanding of this critical human ability. Here we investigated the effects of attention on the perception of bimodal congruent and incongruent face/voice stimuli (see Figure 1) using three gender judgement tasks. Gender discrimination is a common task in unimodal studies, as it requires some depth of processing, but is readily done. In the first task, subjects judged if the gender of the face and the voice were congruent or not. In the second and third tasks subjects categorised the bimodal stimuli by gender, in one case attending only to voices or, conversely, attending only to the faces. The same stimuli were used in the three tasks allowing us to determine effects due only to the task, i.e. top-down influence on the processing of bimodal stimuli. The directed attention aspects of the tasks allowed us to determine the influence of top-down modulation on multimodal processing, whereas the use of congruent and incongruent stimuli provided information on bottom-up stimulus-dependent processing. The use of only congruent and incongruent bimodal stimuli does not allow a direct comparison of responses to bimodal versus unimodal stimuli.

We hypothesized that if vision dominates over audition in gender perception, an incongruent face would disrupt the processing of voice gender while an incongruent voice would have less impact on the perception of face gender. On the other hand, if incongruence has a similar effect regardless of whether subjects performed the task on faces or voices, this would suggest an equivalent influence of the two senses on each other. We also hypothesized that directing attention to one or the other modality would modulate brain activity earlier than stimulus congruency. We showed that directing attention to only one modality modulated early ERPs that were more representative of the attended modality. The congruency task required the processing of both auditory and visual information and the pattern of cerebral activity reflected interaction effects. Comparing congruent and incongruent stimuli allowed us to show that faces dominate over voices in the integration of auditory and visual information of gender, and also demonstrated that bottom-up or automatic processing of the bimodal stimuli arose later (~180 ms) in right frontal regions.

## Results

### Behavioural results

**Figure 2:**
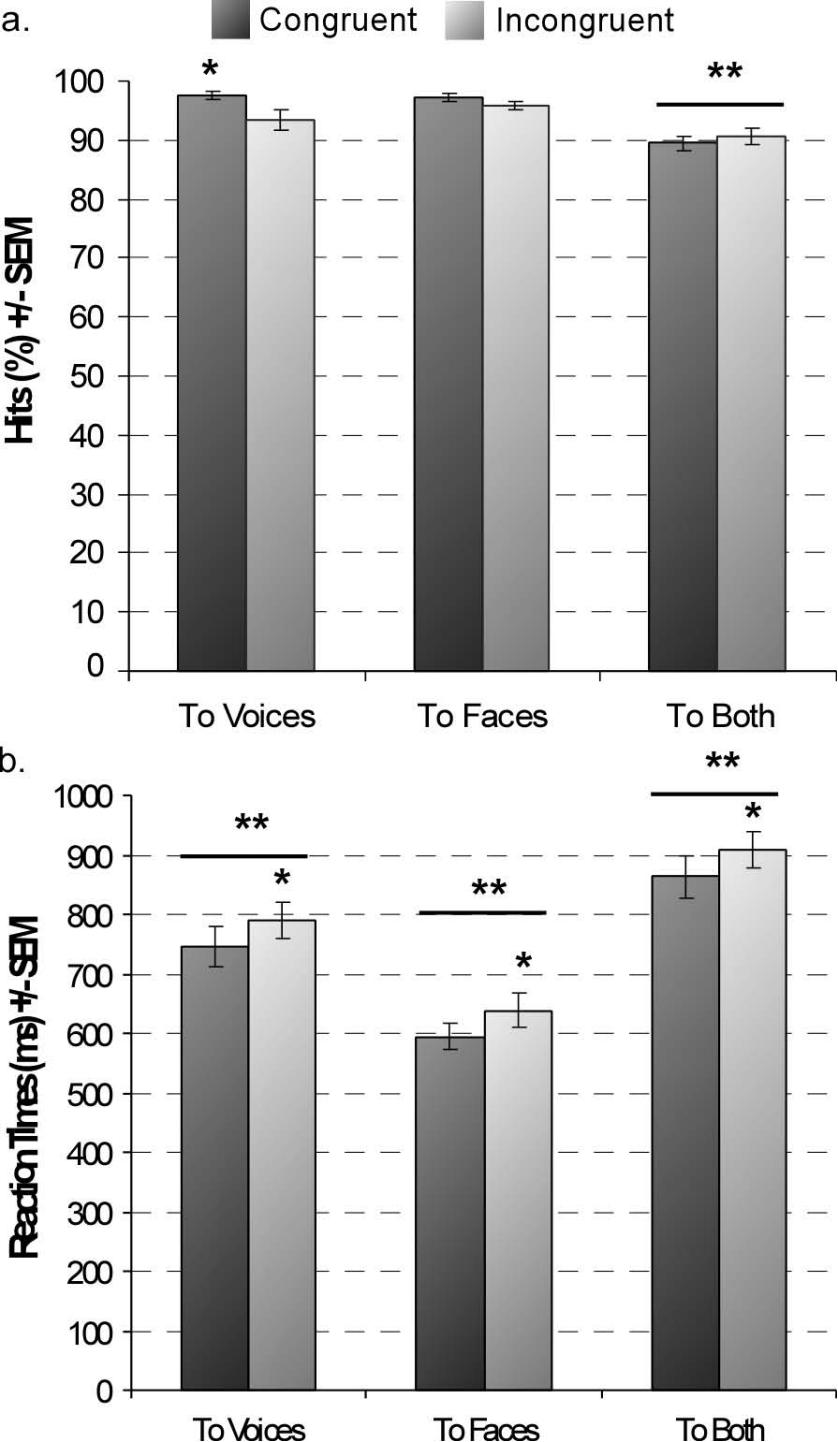
**Behavioural measures**. (**a**) Accuracy for the different tasks. (**b**) Reaction times. Responses to congruent stimuli are in dark and to incongruent stimuli in grey. Greater accuracy was seen for congruent than incongruent stimuli in the VOICE task; overall accuracy in the BOTH task was smaller compared to both FACE and VOICE tasks. Slower RTs were found to incongruent stimuli, regardless of attentional direction. RTs differed significantly across tasks. * p < 0.01; ** p < 0.001

Subjects were equally accurate with gender categorisation of faces (96.47%) and of voices (95.44%); congruency judgement in the BOTH condition was more difficult, reflected by the lower percentage of correct responses ((90.05%) F_2,36 _= 15.96, p < 0.001 - Table 1). Congruency of the face and voice affected gender categorisation performance only during the VOICE task (attention × congruency: F_2,36 _= 7.92, p = 0.002): incongruent face information impaired gender categorisation of voices (congruent: 97.49%; incongruent: 93.38%, difference = 4.11%, 95% CI of the difference = [1.52 7.12]) - see Figure 2a, and Table 1. This impact of incongruent information on subjects' accuracy in the VOICE and not in the FACE task, demonstrated an asymmetry in the processing of faces and voices.

**Table 1:** Hits and Reaction Times for each attentional task and congruence.

	To Voices		To Faces		To Both	
	
	Congruent	Incongruent	Congruent	Incongruent	Congruent	Incongruent
Hits (%)	97.49 ± *0.69*	93.38 ± *1.65*	97.12 ± *0.67*	95.84 ± *0.84*	89.41 ± *1.33*	90.68 ± *1.42*

RTs (ms)	745.68 ± *33.9*	790.13 ± *30*	594.63 ± *23.4*	638.87 ± *28.07*	863.31 ± *35.54*	909.29 ± *31.22*

Reaction times (RTs) were influenced by task (F_2,36 _= 63.09, p < 0.001), being longer in the BOTH task, as the congruency judgment took longer than gender categorisation (paired comparisons, p < .05 - Table 1). Gender categorisation took longer for voices than faces (difference = 151.15 ms, 95% CI = [112.71 191.11], p < .0001 - Figure 2b, Table 1). Finally, incongruent stimuli took longer to categorise for all three tasks regardless of attentional conditions (F_1,18 _= 35.89, p < 0.001 - difference = 44.89 ms, 95% CI = [30.46 59.28]); thus, the bimodal information was processed regardless of whether it was required for the task performance, suggesting an automaticity in face and voice processing.

### Neurophysiological results

**Figure 3:**
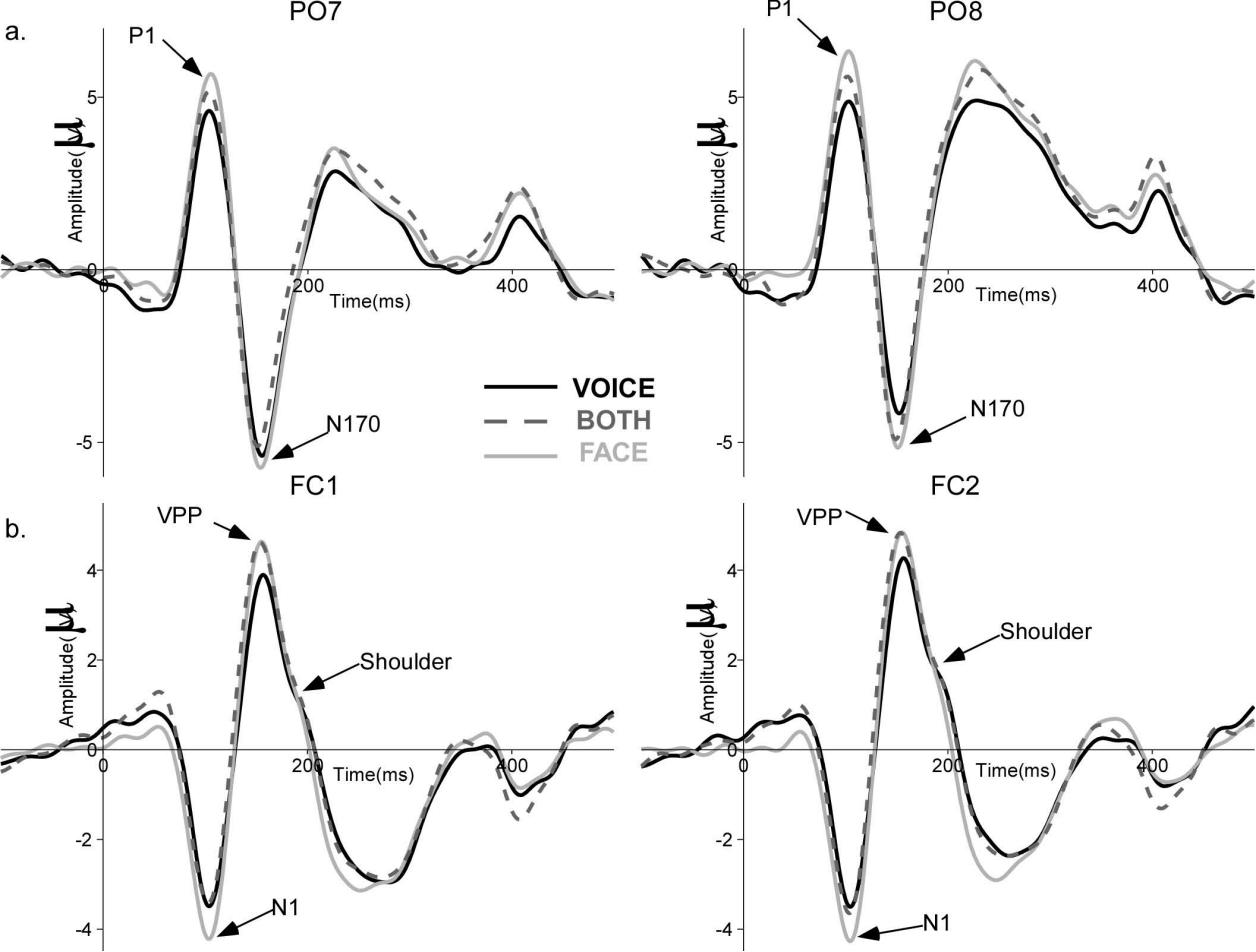
**Grand average ERPs for the three tasks**. (**a**) ERPs at PO7 (left) and PO8 (right) for the congruent stimuli in each attentional task showing the typical P1 and N170 components to faces. (**b**) ERPs at FC1 (left) and FC2 (right) illustrating auditory N1, VPP and the shoulder (likely reflecting the auditory P2) for congruent stimuli in the different tasks. VOICE: solid black line, FACE: solid light grey line, BOTH: dashed dark grey line.

**Figure 4:**
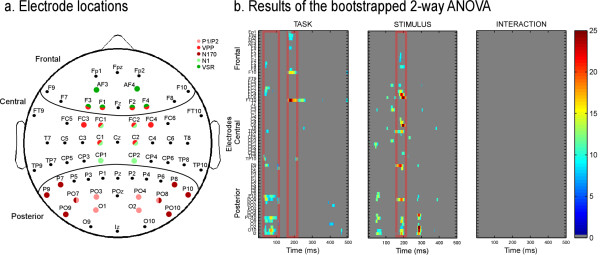
**Results of the bootstrapped ANOVA for the 2 factors and their interaction**. (a) Electrode locations. Red: electrodes on which visual components were measured. Green: electrodes on which auditory components were measured. (b) Results of the bootstrapped 2-way ANOVA. The scale represents F-values, when the 2-way ANOVA was significant after correction for repeated measures, for factor task and stimulus as well as the interaction. Non-significant F-values are presented in grey. Red rectangles indicate latencies of interest, determined by more consistent (spread over several electrodes and time points) and larger effects. This shows both early (30-90 ms) and later (170-220 ms) task effects, stimulus effects at 180-230 ms and no interaction.

Across the three tasks the waveform morphology was similar to that observed in face ERP studies; P1, N170, P2 components recorded from posterior electrodes and N1, VPP, N2 from central electrodes (Figure 3). Spatio-temporal analyses revealed differences in brain activity starting as early as 30 ms after stimulus onset. Task modulated brain activity between 30 and 100 ms and between 160 and 250 ms. Stimuli, i.e. congruency between face and voice, affected brain activity mostly between 150 and 210 ms (see Figure 4b). Both spatio-temporal and peak analyses showed a modulation of brain activity by task and/or stimuli at a number of locations and latency ranges, as detailed below.

### Early effects, P1 and N1 components

P1 amplitude varied with attention as it was larger in the FACE and BOTH tasks than in the VOICE task (F_36,2 _= 8.37, p = 0.001) - Figure 3a. The auditory N1 was larger in the FACE task than in the BOTH and VOICE tasks (F_36,2 _= 4.075, p = 0.029 - Figure 3b). P1 was largest at PO7/PO8 regardless of where attention was directed; however, in the FACE and BOTH conditions, the P1 was second largest at O1/O2, whereas for the VOICE condition P1 at PO7/PO8 and PO3/PO4 were equivalent and larger than at O1/O2 (attention × electrodes: F_72,4 _= 5.25, p = 0.006) (see Table 2). In other words, P1 was largest occipitally in conditions with attention directed to faces. The more anterior topography when subjects attended to voices may reflect overlapping activation of auditory brain areas for early auditory processing. Congruency affected neither P1 (F_18,1 _= 2.357, n.s.) nor N1 (F_18,1 _= 0.378, n.s.) amplitude. Neither P1 nor N1 latencies were affected by attention or congruency (Figure 3).

**Table 2:** P1 amplitude as a function of electrode in the different attentional tasks.

Electrodes	To Voices (μV)	To Faces (μV)	To Both (μV)
O1/O2	4.827 ± *.643*	6.018 ± *.754*	5.431 ± *.808*

PO3/PO4	5.223 ± *.614*	5.667 ± *.697*	4.992 ± *.720*

PO7/PO8	5.349 ± *.642*	6.999 ± *.559*	6.251 ± *.723*

**Figure 5:**
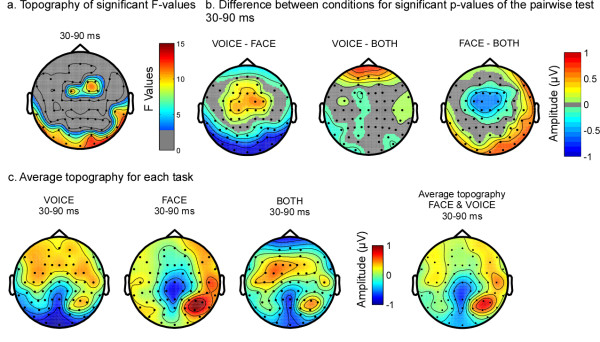
**Attention modulated early brain activity (30-90 ms)**. (a) Topography of the average F-values in this time range. Non-significant F-values are in grey. (b) Topography of the absolute differences between the two tasks where the p-values of the post-hoc test were significant (p < 0.05). Non-significant data are represented in grey. (c) Average topographic maps for each task between 30 and 90 ms. Left to right: FACE, VOICE, BOTH and the average between FACE and VOICE, shown as a comparison. Over posterior regions, the map for the BOTH task is similar to the map for the FACE task, while in fronto-central regions it is more similar to the map for VOICE. Comparison of BOTH with the average of VOICE and FACE shows that the topography in the BOTH task differed from the average topography of the other tasks over fronto-central electrodes.

In the spatio-temporal analyses, early differences were observed over central and posterior temporal brain areas (Figure 4b, 5a) between 30 ms and 90 ms. Post-hoc analyses revealed that the topography differed mostly between the FACE and the VOICE condition (Figure 5b). In Figure 5c, it is evident that the topography for FACE and VOICE are quite distinct and representative of the topography observed for unimodal visual and auditory stimuli at this latency. The topography in the BOTH condition approaches the average of FACE and VOICE topographies in the same latency range (see Figure 5c, far right map); a difference between the BOTH and FACE tasks can be observed over fronto-central regions (Figure 5c, centre two maps).

### N170/VPP

**Figure 6:**
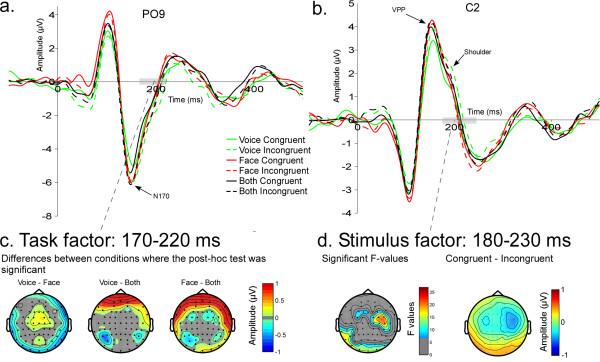
**Task and Stimulus effects between 150 and 250 ms**. N170 (**a**) at PO9 and VPP (**b**) at C2 for the 6 conditions. In green: VOICE task, in red: FACE task, in black: BOTH task. Solid lines: congruent stimuli; dashed lines: incongruent stimuli. **c**) Effects of task between 170 and 220 ms; the two-way ANOVA was significant in frontal regions. Bottom: The maps represent the absolute differences between two conditions where post-hoc tests were significant. Non-significant data are represented in grey. **d**) Modulation of brain activity due to the stimuli between 180 ms and 230 ms for congruent and incongruent stimuli. Left map shows the significant F-values between 180 ms and 230 ms for the factor "stimulus" (non-significant F-values are represented in grey) and the right map shows the difference between topography to congruent and incongruent stimuli (scale: -1 1).

N170 was earlier when attention was directed towards both faces and voices (BOTH - 147.6 ms) than when it was directed towards faces (FACE - 150.7 ms) or voices (VOICE - 155.1 ms) alone (F_36,2 _= 6.93, p = 0.006) (Fig. 6a). N170 latency was shorter in the right hemisphere (RH - 149.9 ms, LH - 152.4 ms; F_18,1 _= 5.25, p = 0.034) (Figure 3a). VPP peaked earlier when attention was directed to faces (154.9 ms) and to both faces and voices (154 ms), relative to when attention was directed only towards voices (160 ms) (F_36,2 _= 4.45, p = 0.04) (Figure 6b). N170 and VPP amplitudes were not significantly affected by task or stimulus (Figure 6a and 6b).

### Later effects; visual and auditory P2s, VSR

Neither attention nor congruency affected the visual P2 or the VSR significantly. Both components showed hemisphere effects, however. The visual P2 was larger in the right than in the left hemisphere (F_1,18 _= 8.54, p = 0.009); the VSR had a shorter latency (F_1,18 _= 10.4, p = 0.005) and larger amplitude (F_1,18 _= 17.42, p = 0.001) over the right hemisphere. The auditory P2 has been proposed to index voice processing [42], yet it was not apparent in our study. We reasoned that the auditory P2 may be masked by the VPP, which occurs in a similar latency range and over the same electrodes.

Spatio-temporal analyses of ERP topography between 170 and 220 ms showed a larger negativity in the BOTH task compared to FACE and VOICE tasks at frontal electrodes (Figure 6c). Post-hoc tests revealed significant differences between VOICE and BOTH on bilateral posterior electrodes as well as differences between VOICE and FACE on bilateral temporal electrodes (Figure 6c). A stimulus-driven congruency effect showed a significantly increased positivity to incongruent stimuli in the P2 latency range between 182 and 230 ms, in right centro-temporal areas associated with an increased negativity in left posterior regions (Figure 6d).

## Discussion

This study investigated the influence of top-down and bottom-up processes on the important human ability of integrating multimodal face/voice stimuli. Top-down influences were manipulated by the task requirements; stimuli were the same in all three tasks, only attentional instructions differed. Bottom-up influences were evident in the processing of congruent versus incongruent stimuli, i.e. how stimulus characteristics influenced the interaction between modalities.

### Top-down and bottom-up influences on behaviour

Behavioural data showed that directing attention toward the auditory or visual modality biased the processing of the bimodal face/voice stimuli. With the same bimodal stimuli in the tasks, we showed that RTs were shorter when attention was directed to faces than voices (regardless of congruency). This is in accordance with other reports studying bimodal natural object recognition [19,27,43] showing that visually based categorisation is faster than auditory based categorisation. RTs were longer for incongruent stimuli regardless of the direction of attention; thus, the unattended modality affected processing in the attended modality, revealing the automatic processing of bimodal information [27]. Incongruent information modulated subjects' accuracy according to the task. Accuracy was lower in the VOICE task when the voice was presented with an incongruent face, an effect not seen in the FACE task.

This result suggests asymmetrical interference between the processing of faces and voices in gender recognition: faces impact the processing of voice gender more than the reverse. A recent study using ambiguous faces showed that low-level auditory features influence the perception of face gender [44]. Although this result could be seen as opposite to ours, this is not the case as the gender of the faces in the Smith et al. study was ambiguous and thus, gender attribution was mostly based on auditory cues. Asymmetrical interference effects have been reported in studies using various paradigms and stimuli, and have been understood as reflecting a sensory dominance in the processing of particular features [8,18,43]. Our results demonstrated that in gender categorisation of faces and voices, visual information dominates auditory information. This dominance of faces over voices for gender discrimination could be explained by different hypotheses of sensory dominance. One is the information reliability hypothesis, which suggests that the dominant modality is whichever is more appropriate and the more efficient for the realisation of the task [45]. In our study the more reliable modality would be vision due to intrinsic properties of the stimuli; information required to perform gender categorisation are easily and immediately extracted from a face, whereas auditory stimuli are always dynamic and thus some number of cycles need to be heard before a voice could be recognised by gender. Another possible hypothesis for the visual dominance would be that sensory dominance results from top-down influences [45]. However, if a stimulus automatically captures attention in one modality (such as faces in the present case), the processing of that stimulus would occur despite attention instructions, and any dominance due to attention would be reduced. This latter explanation is in accordance with studies demonstrating that gender categorisation of faces occurs in the near absence of attention; that gender is automatically extracted from faces [46]. Thus, the automatic processing of faces [47] would reduce or mask the processing in the auditory modality even when attention was explicitly directed to the voices.

The hardest of the three tasks was to determine if the gender of both face and voice was congruent, reflected by this task's lower accuracy and longer RTs. In other multimodal studies, a behavioural facilitation is often reported with bimodal stimuli [1,18,48]. However, in tasks involving identification of a non-redundant target, accuracy is reduced [49] and RTs are generally longer [16]. In the BOTH task, subjects were not identifying a single target but making a congruency judgement which required the extraction of relevant information from both modalities; it is consistent with the literature that this task was the most difficult.

Behavioural results provide evidence of a modulation of the responses by both top-down and bottom-up influences. Bottom-up incongruent information delayed the processing of gender in the attended modality regardless of attention instructions. Top-down processes also impacted gender categorisation of bimodal face/voice stimuli. We suggest that directing attention to a specific sensory modality led to a competition in attentional resources, particularly evident in the VOICE condition. As face processing appears mandatory [50], some attentional resources are automatically allocated to faces, which may account for voice processing being less efficient than face processing with the bimodal stimuli. Directing attention to both auditory and visual modalities (BOTH task) led to longer RTs and lower accuracy, again likely reflecting dispersed attentional resources.

### Top-down and bottom-up influences on ERPs

The ERP waveforms, regardless of the task, were very similar to those described in the face literature [29,32]. This supports the suggestion that in our paradigm face processing dominated over voice processing, in accordance with the conclusions from the behavioural data.

### Modulation of brain activity by top-down processes

Neurophysiological responses were modulated by task as early as 30 ms, as seen in the dissimilar topographies as a function of the direction of attention. Various studies have reported very early activity reflecting bimodal integration when comparing the response to bimodal stimuli to the sum of responses to unimodal stimuli [1,17,18,51]. Early multimodal effects were explained either as anticipatory effects [17] or as recruitment of a novel population of neurons by bimodal stimuli in the visual cortex [1]. In the present study, this early modulation reflected top-down processes, as we found early activation of unisensory cortices of the attended modality attributable to preparatory processes. This is in accordance with fMRI and ERP data showing attention-related modulations in modality-specific cortices for bimodal stimuli [49,52,53]. In the VOICE task, the observed brain topography to the bimodal stimuli showed a larger activity in fronto-central brain regions, whereas in the FACE condition, activity to the bimodal stimuli was larger in right occipital regions. Thus, directed attention to either vision or audition led to greater activation in the respective modality-specific cortices; based indirectly on comparing our results with results in the literature, as we did not use unimodal stimuli. Topography in the BOTH task differed slightly from the average topography of FACE and VOICE condition particularly over fronto-central regions, which might reflect greater attention to voices in the congruency judgment task, as processing voices is less automatic than faces. This is in accordance with the conclusion of the behavioural discussion; directing attention to both faces and voices led to a spread of attention, seen neurophysiologically as an intermediate topography observed for the BOTH task. The early effects in the present study demonstrated that subjects are able to direct their attention to a specific modality; brain activity for the different tasks being representative of the unimodal activity. This is an important finding and justifies the use of paradigms involving directed attention to one sensory modality.

The early visual P1 was larger when attention was directed to faces, seen in FACE and BOTH tasks, consistent with ERP studies showing a larger amplitude for attended versus non-attended stimuli [53]; yet the early auditory N1 amplitude did not show modulation by attention. P1 topography differed across the conditions: P1 in FACE and BOTH was maximal over occipital electrodes whereas P1 in the VOICE task was more parietal. These topographical differences suggested overlapping components affecting the P1 in the VOICE compared to the other two tasks. Furthermore, the three tasks impacted P1 and N1 differently, suggesting a modulation of the N1/P1 complex in central regions by the processing of auditory information. The fronto-central N1 recorded in the present study may be the negative counterpart of the P1, generally observed with visual stimuli [54], or may reflect auditory processing [55]. Unimodal studies of auditory processing find that auditory N1 is enhanced to attended auditory stimuli [56]. The absence of differences on the N1 across the conditions may be due to either a deactivation of auditory cortex when attention was directed to faces or a greater activation of auditory cortex when attention was directed to voices; effects which would cancel each other out, leaving no apparent changes to the bimodal stimuli.

N170 and VPP peaked earlier when attention was directed to both faces and voices (BOTH task), but no amplitude effects were seen. N170 reflects an automatic processing of faces as demonstrated by various studies [57], and its amplitude is not modulated by attention [34,50]; thus, we did not expect a difference in N170 across tasks. In contrast, task affected brain activity around 100 ms, in accordance with studies showing that attention modulates the processing of audiovisual stimuli at different latencies [53].

The auditory P2 was not seen in our data; it probably was obscured by the presence of the VPP. However, we observed a shoulder in the descending slope of the VPP around the auditory P2 latency (180/190 ms [58]) that may correspond to processes normally underlying P2 in unimodal conditions, such as voice processing [42]. Visual inspection of the grand average ERPs revealed that the shoulder was larger in VOICE and BOTH conditions than in the FACE condition; a larger shoulder would imply increased voice processing. In accordance with this suggestion, in the FACE task, the shoulder appeared to be more evident for incongruent stimuli, implying that voices were still processed when they carried incongruent information irrelevant for the task, consistent with the longer RTs in the FACE condition for incongruent stimuli.

The processing of paralinguistic information of faces and voices is shown to be dependent on the sensory modality to which the attention is directed. Moreover, our data showed that the interaction between the processing of faces and voices is asymmetrical with greater influences of visual information than of auditory information. The modulation of bimodal integration by top-down influences could reflect a general mechanism underlying multimodal integration; it is the first time that multimodal ERPs are shown to be task-dependent in the processing of faces and voices at a relatively low-level of processing.

### Modulation of brain activity by bottom-up processes

Congruency affected brain activity between 180 and 230 ms after stimuli onset: incongruent stimuli evoked a more positive activity than congruent stimuli in right anterior frontal regions. fMRI studies using bimodal stimuli have shown that the processing of incongruent and congruent stimuli differed in activation in the inferior frontal gyrus (IFG) and the anterior insula [13,59-61], areas thought to be heteromodal. Activity in these regions decreased for incongruent stimuli [15,62]. The localisation of the modulation of brain activity by congruency in the present study is compatible with the suggestion that differences between congruent and incongruent stimuli arise from insula or right IFG, and provides a latency (190 ms) to the previously described effect in the fMRI literature. This result is also in accordance with other ERP studies that reported differences due to congruency over frontal regions before 200 ms [63]. The inferior frontal gyrus and insula in the left hemisphere are thought to reflect the retrieval and manipulation of linguistic semantic representations [64,65]. Other studies demonstrated the role of right insula and IFG in the detection of asynchrony between auditory and visual stimuli [66]. Our data suggest that those regions could also be involved in more general mismatch judgment such as congruency judgment in terms of gender.

### Limitations

One limitation of the study is the use of natural stimuli that can introduce physical differences between the conditions (e.g. between male and female faces or voices). We were interested in the perception of gender on bimodal face/voice stimuli under normal, ecological conditions; this study allows us to show that using these more natural, less tightly controlled stimuli a bias was observed toward faces in the perception of gender. This result suggests that in everyday life situations the perception of gender from faces will dominate over voices. Further study should investigate the perception of gender on more controlled stimuli: for example by using normalised faces and voices, or by controlling the timbre of individual voices, in order to make the tasks equally difficult across sensory modalities. We believe that this could be assessed by using faces in which all "cultural" cues of gender have been removed and by using vowels instead of words.

Another limitation is the fact that we used only bimodal stimuli. Because we were interested in sensory dominance we did not include unimodal conditions to directly compare responses to bimodal stimuli to responses to unimodal stimuli. It should be noted, first, that the lack of unimodal conditions does not prevent drawing conclusions on the sensory dominance in the perception of voice gender, and second, that the rich literature on both face [29,32,67] and voice perception [41,68,69] allows for at least an indirect comparison with existing studies. Further studies, however, should certainly include unimodal conditions to assess the gain of multimodal information in the perception of voice gender.

## Conclusions

We describe dominance of vision over audition in the perception of voice gender behaviourally and neurophysiologically. We observed that top-down influences modulated the processing of bimodal stimuli as early as 40 ms after stimuli onset, yet this influence depended on the preferential modality for the task, providing evidence for a visual bias in the case of face/voice gender categorisation. This bias may be reversed when studying speech perception - a hypothesis to be validated by further studies. Congruency in face and voice stimuli affected neural responses around 190 ms, suggesting that bottom-up multimodal interactions for gender processing are relatively late.

## Methods

### Subjects

Nineteen English-speaking adults (9 women, range = 20-35 years, mean = 26.4 years) participated in the study. Subjects reported normal medical history and no hearing problems; all had normal or corrected-to-normal vision. They all provided informed written consent; the experiment was approved by the Sunnybrook Health Sciences Research Ethics Board.

### Stimuli and procedure

Stimuli were bimodal auditory/visual stimulus pairs that were front view greyscale pictures of faces, which subtended a visual angle of 8° × 6° (see Figure 1, the face stimuli are published with the consent of the models), associated with a voiced word. Previous studies have reported significant findings with the combination of static faces and voices [27,70]. Face stimuli were photographs of 3 men and 3 women taken while speaking 14 different words, thus a total of 42 female and 42 male faces. Voice stimuli were 14 monosyllabic French words recorded in stereo from 3 female and 3 male speakers; thus, there were also 42 female and 42 male voice stimuli. The words averaged 300 ms in duration, including 10 ms rise and fall times. French words were used with our English-speaking subjects to limit the extent of semantic processing. The voices and faces were randomly associated to form 84 stimuli: 42 were congruent, being female face/female voice and male face/male voice, and 42 were incongruent (i.e., male face/female voice or female face/male voice). Face stimuli were presented for 300 ms in the centre of a computer screen. Auditory stimuli were normalised for intensity using *Matlab*; they were presented binaurally through earphones (*Etymotic Research, Inc.*) at normal speaking levels (68 dB ± 5 dB). Face stimuli onset was synchronised with the onset of auditory stimuli using *Presentation *software; interstimulus intervals varied randomly between 1300 and 1600 ms.

The subjects performed three different gender judgment tasks: 1) The first task was to indicate with one of two keys (right and left ctrl key) whether the stimuli were congruent or incongruent in terms of gender, i.e. the subjects had to pay attention to both face and voice gender (BOTH). Subjects completed two blocks of 84 stimuli; response key attribution was counterbalanced across subjects. As this task differed in terms of response mapping it was always run first. 2) Attention was directed towards the faces: subjects performed a gender discrimination of faces (FACE) while ignoring the voices for 84 trials. 3) In the third task they performed gender discrimination of the voices (VOICE) while ignoring the faces for 84 trials. In the latter two tasks, participants pressed one keyboard key (right and left ctrl key) for female and another for male. The order of the presentation of these two tasks was counterbalanced across subjects, as was the response key attribution.

### EEG recording and analysis

The ERPs were recorded in a dimly lit sound-attenuating booth; participants sat 60 cm from a screen on which stimuli were presented. A fixation cross appeared between presentations and subjects were asked to look at it and refrain from making eye movements. EEG was recorded using an ANT (*Advanced Neuro-Technology, Enschede, Netherlands*) system and a 64 electrode cap, including three ocular electrodes to monitor vertical and horizontal eye movements. Impedances were kept below 5 kΩ. The sampling acquisition rate was 1024 Hz. FCz was the reference during acquisition; an average reference was calculated off-line.

Continuous EEG was epoched into 600 ms sweeps including a 100 ms pre-stimulus baseline. Ocular and muscular artefacts, or trials containing an amplitude shift greater than 100 μV, were rejected from analyses. Epochs were averaged by condition (6 conditions: congruent/incongruent in the 3 tasks) and filtered using a bandpass filter of 1-30 Hz.

Peak analyses were completed on the classical peaks described in the visual, i.e. P1, N170, P2 and VPP (Vertex Positive Potential - [71]), and the auditory ERP literature, i.e. N1, VSR [39]. Unimodal auditory stimuli generally evoke biphasic ERPs, the negative N1, mentioned above, followed by the auditory P2 in fronto-central regions, a positive wave occurring between 160 and 240 ms after stimulus onset [58]. An auditory P2 was not seen in our data probably due to its temporal coincidence with the VPP, thus being masked by the VPP. Peak latencies and amplitudes were measured for each participant in a ± 30 ms time-window centred on the latencies of the peak in the grand average (visual - P1: 105 ms, N170: 155 ms, VPP: 160 ms and P2: 220 ms; auditory - N1: 100 ms and VSR: 350 ms, see Figure 3). P1 and P2 were measured at O1/O2, PO7/PO8 and PO3/PO4. N170 was measured at PO9/PO10, PO7/PO8, P7/P8 and P9/P10. VPP was measured at FC1/FC2, FC3/FC4, F1/F2, F3/F4 and C1/C2. Auditory N1 was measured at FC1/FC2, C1/C2 and CP1/CP2, and VSR at AF3/AF4, F3/F4 and F1/F2 (see Figure 4a). Latencies were measured at one time point per hemisphere at the electrode with the largest amplitude. Amplitudes were taken at this latency at the other selected electrodes over the hemisphere [72].

Peak analyses have been extensively used in ERP literature; however, this technique restrains the analysis to time intervals where a peak is seen. In contrast, spatio-temporal analyses determine when brain activity differs significantly between two conditions and allows ERP differences to be identified independently of peak measures [1,16]. Studies of multimodal processing have shown early modulation of brain activity around 40 ms [1,73] that does not correspond to a precise peak. Thus, we also analysed spatio-temporal effects by comparing brain activity at each time point and electrode.

### Statistical analyses

Behavioural data and peak latencies and amplitudes were submitted to repeated measures analyses of variance (using SPSS11); within subject factors were task (3 levels), stimulus (2 levels) and hemisphere (2 levels) for peak latencies plus electrode (different levels depending on the component) for peak amplitudes. After main effects were assessed, we performed paired comparison and post-hoc tests (for interactions) to determine the factors leading to the effects.

Spatio-temporal effects were assessed by comparing brain activity for the different conditions, at each time point and electrode. Repeated measures ANOVA within the general linear model framework were run on the ERPs using *Matlab7.2 *with task and stimulus as inter-subject factors at each time point and electrode. To estimate the statistical significance of the ANOVA, we calculated a data-driven distribution of F-values using a bootstrap-F method; this method makes no assumption on the normality of the data distribution and is therefore robust to normality violations [74,75]. Data were centred at 0 to be under the null hypothesis that conditions do not differ from 0. ANOVAs at each time point and electrode were run on the centred data after resampling the subjects with replacement. We stored the bootstrapped F-values for each time point and electrode independently. This operation was repeated 999 times to obtain a distribution of 1000 bootstrapped estimates of F-values under the null hypothesis [74]. To correct for multiple comparison, we stored the maximum F-values obtained across all time points in each random sampling loop and for each electrode independently [76]. We then calculated a 95% confidence interval of the maximum F-values for each electrode. The repeated measures ANOVA was considered significant if the F-value fell outside the bootstrapped 95% confidence intervals for each time point and electrode (Degrees of freedom (df) are similar for all statistics presented in this study: 2 and 36 for the task factor, 1 and 18 for the stimulus factor and 2 and 36 for the interaction (df of factor and error respectively)).

Post-hoc tests were run for the Task factor whenever the ANOVA was significant. Data-driven confidence intervals were calculated for each comparison (VOICE vs. FACE, VOICE vs. BOTH and FACE vs. BOTH). We performed the analyses across subjects by sampling conditions with replacement (electrodes by time points matrices), independently for each subject. For each random sample, we averaged ERPs across subjects independently for each condition, then computed the difference between the averages for the two conditions (for instance VOICE vs. FACE). In each random sampling loop and for each electrode independently, we stored the maximum absolute difference obtained across all time points. This process was repeated 1000 times, leading to a distribution of bootstrapped estimates of the maximum absolute difference between two ERP conditions, averaged across subjects, under the null hypothesis H0 that the two conditions were sampled from populations with similar means. Then the 95% confidence interval of the mean maximum absolute differences was computed at each electrode (alpha = 0.05). Finally, absolute differences between two sample means at any time point at one electrode were considered significant if they fell outside the H0 95% confidence interval for that electrode.

## Authors' contributions

ML designed the experiment, recorded and analysed the data and wrote the manuscript. RVR helped in analysing the data and writing and reading the manuscript, MJT helped in designing the experiments, writing and reading the manuscript. All authors read and approved the final manuscript.
